# 813. Anatomical Site Impacts Electrical Burn Outcomes

**DOI:** 10.1093/jbcr/irag033.252

**Published:** 2026-04-06

**Authors:** Mare G Kaulakis, Simon Rodriguez, Christopher J Fedor, Sarah M Tepe, Francesco M Egro

**Affiliations:** University of Pittsburgh Medical Center, Mercy Burn Center, Pittsburgh, PA; University of Pittsburgh School of Medicine, Pittsburgh, PA; University of Pittsburgh Medical Center, Department of Plastic Surgery, Pittsburgh, PA; University of Pittsburgh Medical Center, Pittsburgh, PA; University of Pittsburgh Medical Center, Pittsburgh, PA

## Abstract

**Introduction:**

Patients who receive burns from electrical injuries are at a greater risk for several negative clinical course outcomes, such as increased length of stay, risk of amputation, and infection. This study is among the largest cohorts of electrical burns and explores which clinical outcomes are most impacted by anatomical site.

**Methods:**

Using data from the Burn Care Quality Platform (BCQP) Registry from 2013 to 2022, a retrospective review was conducted on patients with electrical burns. In addition to anatomic site, demographics, occupational and situational risk factors, social determinants, and clinical course metrics were analyzed. Data were reported using descriptive and comparative statistics.

**Results:**

7586 patients (mean age 35.4 ± 16.9, 88.4% male) had electrical burn injuries and were included. These burns were primarily caused by accidental employment-related injuries (n = 4268, 56.94%). The most affected body parts were the hand (n = 2679, 65.8%), lower arm (n = 1289, 31.4%), torso (n = 1021, 24.9%). 4589 (60.7%) of the electrical burn injury patients required surgery. Of these patients, hand involvement resulted in higher rates of skin graft loss (0.91% vs 0.11%, *p*<.001). Electrical burns involving the foot resulted in wound infection more frequently (1.89% vs 0.65%, *p*=.002). There were no differences in acute surgical complications by burn location (all *p*>.05). Based on our multiple linear regression, hospital length of stay was significantly increased for patients with torso (β = 3.96, *p*<.001) and lower extremity injuries, particularly the foot (β = 4.8, *p*<.001).

**Conclusions:**

Patients with electrical burn injuries have varying clinical outcomes depending on the location of the burn. In particular, electrical burns of the extremities have the most complications, with hand burns leading to increased graft loss and foot injuries being correlated with increased rates of infection and longer lengths of stay. These findings add to the literature on clinical outcomes in electrical burns.

**Applicability of Research to Practice:**

This study provides information on the locations of electrical burns that are at particularly high risk for complications. By doing so, clinicians can give these high-risk patients increased attention and resources to optimize their treatment outcomes.

**Funding for the study:**

N/A.

